# Mothers' Awareness of Obesity and Its Complications Among Children in the Western Region of Saudi Arabia: A Cross-Sectional Study

**DOI:** 10.7759/cureus.57505

**Published:** 2024-04-03

**Authors:** Sultan Almalki, Shadi Tamur, Ahmad Alzahrani, Maryam S Aljaid, Anwar Shams, Maram Alayli, Asrar Alrabie, Abdullah M Khayat

**Affiliations:** 1 Department of Pediatrics, College of Medicine, Taif University, Taif, SAU; 2 Department of Pharmacology, College of Medicine, Taif University, Taif, SAU; 3 Medical School, Taif University, Taif, SAU

**Keywords:** pediatric healthcare, parents' awareness, nutrition and exercise, prevention strategies, risk factors, active lifestyle, diabetes, obesity

## Abstract

Background: Awareness about obesity, its complications, and an age-appropriate, healthy, active lifestyle are essential to maintaining a healthy life. This study aimed to determine the level of awareness Saudi Arabian caregivers have regarding obesity and regular physical activity.

Materials and methods: In this cross-sectional study, a survey was conducted among parents in Saudi Arabia using a structured questionnaire. A convenience and snowball sampling method was employed to recruit participants from various regions of Saudi Arabia. The questionnaire assessed parents' knowledge about obesity, its complications, and healthy, active lifestyle practices. Data were analyzed using descriptive statistics, and associations between variables were examined using chi-squared tests.

Results: In this study, responses from 256 moms in various Saudi Arabian locations were included in the analysis. Merely 35.5% of the participants demonstrated good knowledge about obesity and its complications. Regarding the promotion of a healthy, active lifestyle, only 11.3% of the participants were aware that kids should eat five types of vegetables and fruits daily, whereas 85% were aware that kids should have <2 hours a day of screen time, and 75.4% were aware that kids should be engaged in physical activity for at least one hour daily.

Conclusion: This study highlights a concerning gap in knowledge among caregivers in Saudi Arabia concerning obesity and its consequences, with merely 35.5% of participants demonstrating a good understanding of obesity and its complications. Alarmingly, only a fraction (11.3%) showed awareness of the importance of promoting a healthy, active lifestyle. These findings underscore the urgent need for enhanced awareness initiatives focused on obesity and its prevention to ensure optimal child health development, avert health issues, and strengthen positive dynamics between parents and children.

## Introduction

Obesity is a serious medical condition worldwide and requires new approaches and recognized international consensus in treating related diseases [1]. Worldwide obesity rates have increased exponentially in recent decades. The rapid rise in obesity is a major concern for global public health [2]. The escalating global obesity epidemic has seen a dramatic surge from 1975 to 2016, with obesity rates nearly tripling worldwide. The World Health Organization (WHO) has highlighted this concerning trend, noting that in 2016, the number of overweight or obese children and teenagers aged 5-19 exceeded 340 million. This represents a significant increase from a mere 4% in 1975 to over 18% in 2016, indicating a widespread and growing issue among young populations. The rise in obesity rates was consistent across gender lines, with 19% of boys and 18% of girls classified as overweight in 2016 [3]. Furthermore, the WHO's findings reveal that the problem extends to even younger demographics, with 39 million children under five years old being identified as obese or overweight in 2020 [3]. Unequivocally, global obesity rates have increased exponentially in recent decades. Younger people are becoming obese, morbid obesity rates are increasing, and the full health implications are only beginning to be seen [4].

Over the past 20 years, there has been a significant rise in the proportion of overweight and obese children and adolescents in Saudi Arabia, which has sparked worries about the psychological and physical effects of childhood obesity. Compared with the rest of the world, in Saudi Arabia, the percentage of obese males is close to 20% for those aged 5-9 and 24% for those aged 10-14, while among Saudi females, the prevalence of obesity is 40% between the ages of 5 and 9 and 41% between 10 and 14 [5].

The best population-level indicator of overweight and obesity is the body mass index (BMI), calculated by weight per kg divided by height in meters squared, for all children aged 5-19 and for both sexes. Therefore, for children aged 5-19, overweight is defined as having a BMI that is more than 1 standard deviation over the WHO Growth Chart median, while obesity is defined as having a BMI that is more than 2 standard deviations above the WHO Growth Chart median [2]. On the other hand, for children under the age of five, obesity is better defined as weight-for-height greater than 3 standard deviations above the WHO Child Growth Standards median, while overweight is defined as weight-for-height greater than 2 standard deviations above the WHO Child Growth Standards median [2].

Childhood obesity is a result of an interplay of genetics, environment, and behavior [4]. In children, the most common cause of obesity is a positive energy imbalance caused by caloric intake greater than caloric expenditure combined with a genetic predisposition for weight gain [5]. Most studies have focused on biological risk factors, which can be broadly categorized as genetic predisposition, poor diet (and behaviors that influence excessive food intake), insufficient physical activity, and developmental factors in early life that influence long-term health [3]. Endocrine etiologies for obesity are rare and are usually accompanied by attenuated growth patterns [6].

The increasing prevalence of childhood obesity is associated with the emergence of comorbidities previously considered "adult" diseases, such as type 2 diabetes mellitus, hypertension, non-alcoholic fatty liver disease, obstructive sleep apnea, and dyslipidemia [7]. Childhood obesity is also linked to an increased risk of obesity and early mortality in adulthood. Moreover, obesity in children is associated with higher risks in the future for cardiovascular disease, insulin resistance, and psychological consequences [8]. Childhood obesity also increases the likelihood of developing cancer and autoimmune diseases. In one study, the premature mortality rate was estimated to be three times higher before 30 years of age in children with obesity than in the normal population [9].

The noncommunicable diseases associated with overweight and obesity (e.g., diabetes, hypertension, and dyslipidemia) are mostly preventable. To avoid overweight and obesity, supportive settings and communities play a crucial role in influencing people's decisions by making the selection of healthy foods and regular physical activity the most straightforward (i.e., the option that is most readily available, affordable, and accessible). Besides, research has indicated that exclusively breastfeeding a baby from birth to six months of age lowers the likelihood that the baby will grow up to be overweight or obese [8].

In order to decrease and prevent childhood overweight and obesity, the WHO suggests the following: limit energy intake from total fats and shift fat consumption from saturated fats to unsaturated fats; limit the intake of sugars; and engage in frequent, developmentally appropriate, moderate-to-intense physical activity for at least 60 minutes each day [9]. Long-term outcomes depend on the involvement of the government in creating policies that support the creation of an environment and opportunity for a healthy diet and physical activity, as well as the involvement of the family, school, and community [9].

Three main elements should be included in every treatment plan for obese children and adolescents: family-based behavior management, diet control, and daily exercising [7]. Restrictive diets shouldn't be implemented for kids or teenagers because healthy growth and development require a sufficient amount of calories. It is also imperative that any program or treatment plan consider the caregivers, who are typically obese or overweight. Treatment with anti-obesity medications is not advised for use in younger children and has a restricted role in childhood. Although there are few long-term safety data on bariatric surgery in older adolescents who are extremely obese or have other comorbidities including hyperlipidemia, non-alcoholic liver disease, type 2 diabetes, and cardiovascular disease, the procedure is still reserved for them [10]. Evidently, it has been determined that childhood obesity is a worldwide pandemic. Mothers often play a central role in dietary choices and physical activity levels in the household, making their awareness pivotal in forming healthy habits early on. Additionally, understanding the level of awareness helps in identifying knowledge gaps and misconceptions that may hinder effective prevention and management of child obesity, allowing for more focused and effective communication strategies. The aim of our study is to assess mothers' knowledge and awareness about obesity and its complications. Therefore, by assessing this awareness, public health policies can be better designed to support families in creating environments that promote healthy growth and development, reducing the long-term health complications associated with childhood obesity. 

## Materials and methods

Methods and data analysis

A structured questionnaire with informed consent was used to conduct a survey among Saudi Arabian parents as part of this cross-sectional study (see Appendices). The study utilized a convenience and snowball sampling technique to include participants (mothers) from different ages and diverse regions within the western region of Saudi Arabia. Using an online survey, the purpose of the questionnaire was to gauge mothers' understanding of healthy active lifestyle practices, obesity risk factors, and its complications.

To ensure the accuracy, reliability, and validity of the study findings, a comprehensive data analysis and data management plan were implemented. Data cleaning and preprocessing were performed to ensure the quality and integrity of the dataset. This involved identifying and addressing any missing, incomplete, or erroneous data. Outliers were examined and treated appropriately to minimize their effect on the analysis. In addition, data coding and categorization were performed to facilitate subsequent analysis. IBM SPSS Statistics for Windows, Version 25.0 (Released 2017; IBM Corp., Armonk, New York, United States) was used for data analysis. Descriptive statistics were employed to summarize and describe the key characteristics of the variables of interest. Inferential statistics, such as chi-squared tests, were used to examine relationships, test hypotheses, and identify significant associations between variables.

## Results

The analysis included responses from 256 mothers from different regions of Saudi Arabia. Regarding the characteristics of the mothers, 79 (30.9%) had 3-4 children, 101 (39.5%) belonged to the group aged 41-50 years, 244 (95.3%) were married, 156 (60.9%) had an experience of being a mother for >10 years, and 146 (57%) had education at the graduate level (Table 1).

**Table 1:** Sociodemographic characteristics of the participants

Sociodemographic characteristics	Participant response items	n	%
Number of kids	1	45	17.6
	2	52	20.3
	3-4	79	30.9
	4-5	44	17.2
	>5	36	14.1
Age of the mother	20-30 years	61	23.8
	31-40 years	94	36.7
	41-50 years	101	39.5
Experience as mother	1-3 years	42	16.4
	3-5 years	27	10.5
	5-10 years	31	12.1
	>10 years	156	60.9
Marital status	Married	244	95.3
	Divorced	12	4.7
Educational level	Illiteracy	2	.8
	Primary	12	4.7
	Middle school	15	5.9
	Secondary	66	25.8
	Postgraduate	15	5.9
	Graduate	146	57.0

Moreover, 14 items in the questionnaire were related to awareness of obesity, and the responses of the participants are given in Table 2. The majority (94.9%) agreed that obesity is considered a disease, and approximately 98.4% agreed that obesity can lead to complications such as diabetes, hypertension, and respiratory disorders. Approximately 70.3% agreed that a relationship exists between obesity and asthma, and 84.4% agreed that obesity affects the growth and development of the body of the child. The majority of the mothers (98.8%) agreed that physical activity and daily sports reduce obesity, and approximately 75.8% had the view that physical activity reduces the risk of obesity complications such as diabetes mellitus and heart diseases.

**Table 2:** Responses to knowledge and awareness items

Knowledge items	Responses	n	%
Is obesity considered a disease?	No	13	5.1
	Yes*	243	94.9
Complications of obesity (diabetes/pressure/respiratory problems)	No	4	1.6
	Yes*	252	98.4
Is there a relationship between obesity and asthma?	No	76	29.7
	Yes*	180	70.3
Does obesity affect the growth and development of the body of the child?	No	40	15.6
	Yes*	216	84.4
Does physical activity and daily sports reduce obesity?	No	3	1.2
	Yes*	253	98.8
Exercising reduces a child's risk of obesity complications such as:	Diabetes	46	18.0
	Heart disease	16	6.3
	All of the above*	194	75.8
Children should engage in sports or physical activity at least:	1 h a day*	193	75.4
	2 h a week	28	10.9
	4 h a week	35	13.7
How much time can a child watch television and play electronic games per day?	<2 h*	219	85.5
	>5 h	1	.4
	3-5 h	34	13.3
	5-8 h	2	.8
When you sit for >1 hour, it is recommended to:	Move and do physical activity for 1 min every 2 h	22	8.6
	Move and do physical activity for 3 min every half hour*	67	26.2
	Move and do physical activity for 5 min every hour	167	65.2
The most important meal that helps reduce obesity and promote a healthy lifestyle is:	Breakfast*	195	76.2
	Lunch	13	5.1
	Dinner	27	10.5
	Snacks	21	8.2
How many types of fruits and vegetables should the child eat per day?	2 types	117	45.7
	3 types	100	39.1
	5 types*	29	11.3
	7 types	10	3.9
The child is advised to eat grains and legumes:	With breakfast daily	132	51.6
	With lunch daily	60	23.4
	With every meal 4 times a week*	64	25.0
Eating fish reduces the risk of heart disease and promotes brain health. How often should the child eat it?	Daily	14	5.5
	2 days a week*	212	82.8
	4 days a week	30	11.7
The permissible limit for drinking sweetened beverages is:	5 times a week	1	.4
	Twice a week	28	10.9
	Once a day	6	2.3
	Not allowed*	221	86.3

Furthermore, a majority of 85.5% of mothers permitted their kids to watch television and play video games for less than two hours every day. Only 26.2% of mothers agreed that their children move and do physical activity for three minutes every half hour. Approximately 76.2% agreed that breakfast is the most important meal that helps reduce obesity and promote a healthy lifestyle, and only 11.3% agreed that a child should eat five types of fruits and vegetables per day. Only one-fourth (25%) agreed that children are advised to eat grains and legumes. However, a majority (82.8%) agreed that eating fish twice a week reduces the risk of heart disease and promotes brain health. Approximately 86.3% agreed that children are not allowed to drink sweetened drinks; however, approximately 10.9% said it could be allowed twice a week.

**Figure 1:**
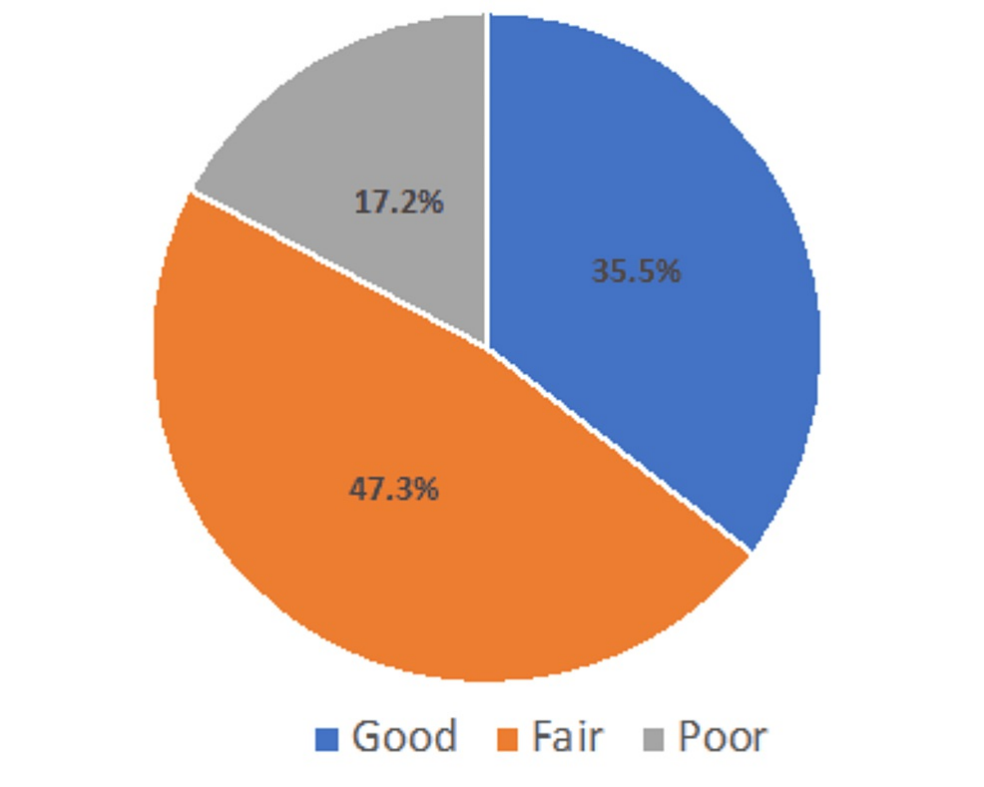
Mothers' knowledge levels related to obesity

The responses to each awareness question were categorized as correct and wrong, and scores were given accordingly. The total awareness score was 14, and the minimum was 0. The total scores were converted into percentages and then further classified into three awareness levels: good (≥75%), fair (60-74.9%), and poor (<60%). Approximately 35.5% had good, 47.3% had fair, and 17.2% had poor awareness levels (Figure 1).

The results of the assessment of awareness levels and different sociodemographic characteristics are presented in Table 3. No statistically significant relationship was observed between awareness levels and any of the sociodemographic characteristics (p>0.05).

**Table 3:** Relationship between knowledge level and sociodemographic characteristics

Sociodemographic characteristics			Knowledge level			Total	P-value
			Good	Fair	Poor		
Number of kids	1	N	23	15	7	45	0.382
		%	51.1%	33.3%	15.6%	100.0%	
	2	N	17	25	10	52	
		%	32.7%	48.1%	19.2%	100.0%	
	3-4	N	23	43	13	79	
		%	29.1%	54.4%	16.5%	100.0%	
	4-5	N	18	19	7	44	
		%	40.9%	43.2%	15.9%	100.0%	
	>5	N	10	19	7	36	
		%	27.8%	52.8%	19.4%	100.0%	
The age of the mother	20-30 years	N	23	25	13	61	0.766
		%	37.7%	41.0%	21.3%	100.0%	
	31-40 years	N	32	48	14	94	
		%	34.0%	51.1%	14.9%	100.0%	
	41-50 years	N	36	48	17	101	
		%	35.6%	47.5%	16.8%	100.0%	
How many years of experience do you have as a mother?	1-3 years	N	17	15	10	42	0.147
		%	40.5%	35.7%	23.8%	100.0%	
	3-5 years	N	10	14	3	27	
		%	37.0%	51.9%	11.1%	100.0%	
	5-10 years	N	15	15	1	31	
		%	48.4%	48.4%	3.2%	100.0%	
	>10 years	N	49	77	30	156	
		%	31.4%	49.4%	19.2%	100.0%	
Marital status	Married	N	89	113	42	244	0.322
		%	36.5%	46.3%	17.2%	100.0%	
	Unmarried	N	2	8	2	12	
		%	16.7%	66.7%	16.7%	100.0%	
Educational level	Illiteracy	N	1	0	1	2	0.754
		%	50.0%	0.0%	50.0%	100.0%	
	Primary	N	5	4	3	12	
		%	41.7%	33.3%	25.0%	100.0%	
	Middle school	N	7	5	3	15	
		%	46.7%	33.3%	20.0%	100.0%	
	Secondary	N	19	36	11	66	
		%	28.8%	54.5%	16.7%	100.0%	
	Graduate	N	53	70	23	146	
		%	36.3%	47.9%	15.8%	100.0%	
	Postgraduate	N	6	6	3	15	
		%	40.0%	40.0%	20.0%	100.0%	

## Discussion

Childhood obesity is a growing global concern with profound implications for immediate- and long-term health outcomes. Mothers play pivotal roles in shaping their children's lifestyles and overall health [11]. Childhood obesity refers to the excessive accumulation of body fat in children and adolescents. It is typically assessed using the BMI percentile for age and sex. A BMI at or above the 95th percentile is considered obese, whereas a BMI between the 85th and 95th percentiles is classified as overweight [12]. The prevalence of childhood obesity in Saudi Arabia, as per recent evidence, shows alarming findings, where 11.2% and 9.4% were overweight and obese, respectively [13]. In this study, mothers have moderately good awareness levels regarding obesity. The findings of previous studies that mothers had good awareness related to childhood obesity despite the high prevalence of obesity [13,14] suggest a potential gap between knowledge and practical implementation. While mothers have a good theoretical understanding of obesity and its consequences, they might face challenges translating this knowledge into practical behavioral changes for their children. Factors such as convenience, cultural norms, and socioeconomic constraints can influence their ability to implement healthy habits consistently [15-17].

The causes of childhood obesity are multifaceted and complex, including genetics, socioeconomic status (SES), race, ethnicity, sex, lifestyle, and diet [18-20]. However, our findings did not show any relationship between maternal characteristics and knowledge level. Several maternal characteristics, such as the mother's age, education level, and family income, may influence their knowledge levels. However, discussions about these aspects are needed [21]. A mother's understanding of childhood obesity and its potential health effects is essential to promoting their children's well-being. Mothers can actively contribute to the prevention of obesity and its health concerns by being aware of the short- and long-term effects of obesity on their children's health. Mothers can substantially influence their children's long-term health through healthy lifestyle choices and nurturing circumstances [22,23]. The WHO, American Medical Association, and World Obesity Federation classify obesity as a chronic medical condition and disease [24]. In this study, most of the mothers considered obesity a disease. Obesity-associated comorbidities have also been on the rise, mirroring the epidemic of childhood obesity. Many studies have demonstrated a strong association between obesity and serious health conditions, such as type 2 diabetes mellitus, cardiovascular diseases, certain cancers, sleep apnea, and fatty liver disease [25-28]. The risk of developing these diseases increases with the severity of obesity. Some argue that obesity is a complex condition influenced by individual behaviors, genetics, environment, and societal factors [21,29-30]. They suggest that labeling it as a disease might lead to overlooking the issue and undermine personal responsibility for healthy choices.

Previous studies have suggested that in countries with lower economic status, the detrimental effect of increased maternal education on children's physical activity is more pronounced than a higher level of paternal education [31-34]. Hypothetically, this disparity could result from mothers in economically underdeveloped nations having greater access to motorized transportation due to their higher socioeconomic status and higher levels of education. Additionally, these mothers are typically more responsible for determining the modes of transportation used by their children.

The findings of previous studies exploring the relationship between maternal education and childhood obesity have been inconclusive. Some researchers have linked mothers with higher education levels to children who are overweight [35]. By contrast, another study found that parents with a college education could help prevent their children from becoming overweight [36]. Likewise, another study showed that mothers with higher education might be more likely to feed their children healthy, well-balanced meals, which has been linked to a decrease in childhood obesity [37]. Maternal parenting styles, such as being overly controlling or using food as a reward, can influence a child's relationship with food and eating behaviors.

Limitations

The study's sample might only represent some of the maternal population. Those who are more health conscious or have higher levels of education might be more likely to participate, leading to skewed results. Participants might be influenced by their perceived role or social expectations as mothers; as a result, societal pressures may influence responses. Participants might provide socially desirable responses, leading to an overestimation of their knowledge about obesity to appear more informed or health conscious. Participants might need help recalling and reporting accurately, potentially leading to inconsistencies in their responses. Studies using questionnaire surveys might not capture the nuances or depth of mothers' understanding about obesity, as open-ended questions or interviews might be more suitable for in-depth exploration. Mothers might answer questions that align with societal expectations or perceived norms rather than reflect their actual knowledge or beliefs. To address these limitations, researchers could consider using a mixed-methods approach, conducting follow-up interviews to gain deeper insights, and validating the questionnaire to ensure its comprehensibility and reliability.

## Conclusions

This study underscores a critical knowledge gap among caregivers in Saudi Arabia regarding the consequences of obesity, with just 35.5% of participants exhibiting a solid understanding of obesity and its complications. Alarmingly, a mere 11.3% were aware of the significance of encouraging a healthy, active lifestyle among children. On a positive note, a substantial majority (85%) acknowledged the need to limit screen time. These findings highlight the imperative role of the Ministry of Health in spearheading awareness campaigns aimed at educating mothers and caregivers about obesity prevention and the promotion of healthier lifestyles. Such initiatives are crucial for fostering optimal child health development, preventing health problems, and enhancing the parent-child relationship.

## Appendices

Survey on measuring mothers' awareness and knowledge of obesity and its complications among children in the western region of Saudi Arabia

We are a research team from Taif University conducting a study to measure mothers' awareness of obesity and its complications among children in the western region of the Kingdom of Saudi Arabia.

We invite you to participate in this study. Your participation is voluntary, and your information will be used for scientific research purposes only and will remain completely confidential.

1. Do you agree to participate in the research? 

- Yes                        

- No

2. How many children do you have?

- One child

- Two children

- 3-4 children

- 4-5 children

- More than five children

3. How old are you? 

- 20-30 years

- 31-40 years

- 41-50 years

4. How many years of experience do you have as a mother?

- 1-3 years

- 3-5 years

- 5-10 years

- More than 10 years

5. What is your marital status?

- Married

- Not married (divorced or widowed)

6. What is your educational level?

- Illiterate

- Primary

- Intermediate

- University

- Postgraduate studies

7. Is obesity considered a disease?

- Yes

- No

8. Are diabetes, high blood pressure, and respiratory problems complications of obesity?

- Yes

- No

9. Is there a relationship between obesity and asthma?

- Yes

- No

10. Does obesity affect the growth and development of a child's body?

- Yes

- No

11. Do physical activity and daily sports reduce obesity?

- Yes

- No

12. Child exercise reduces the risk of obesity complications, such as:

- Heart diseases

- Diabetes

- Some types of cancer

- All of the above

13. A child should practice sports or physical activity at least:

- An hour a day

- Two hours a week

- Four hours a week

14. How much time is allowed for a child to spend in front of TV screens and electronic games per day?

- Less than two hours

- 3-5 hours

- 5-7 hours

- More than that

15. When your child spends more time sitting, it is advised to:

- Move and do physical activity for five minutes every hour

- Move and do physical activity for three minutes every half-hour

- Move and do physical activity for a minute every two hours

16. The most important meal that helps reduce obesity and promotes a healthy lifestyle pattern is:

- Breakfast

- Lunch

- Dinner

- Snacks

17. How many types of fruits and vegetables should a child consume daily?

- Two types

- Three types

- Five types

- Seven types

18. Children are advised to consume grains and legumes.

- With breakfast daily

- With lunch daily

- With all meals four times a week

- Eating fish reduces the risk of heart disease and promotes brain health

19. How often should a child consume it?

- Daily

- Two days a week

- Four days a week

- Once a day

- Twice a week

- Five times a week

20. The allowed limit for drinking sweetened beverages like Pepsi:

- Not allowed

- Once daily

- Twice a week

- Five times a week
